# Risk factors of peripheral arterial disease: a case control study in Sri Lanka

**DOI:** 10.1186/s13104-016-2314-x

**Published:** 2016-12-09

**Authors:** Janaka Weragoda, Rohini Seneviratne, Manuj C. Weerasinghe, SM Wijeyaratne

**Affiliations:** 1 Ministry of Health, Baddegama wimalawansa Mawatha, Colombo 10, Sri Lanka; 2 Department of Community Medicine, Faculty of Medicine, University of Colombo, Colombo, Sri Lanka; 3 Department of Surgery, Faculty of Medicine, University of Colombo, Colombo, Sri Lanka; 4 Department of Nutrition Sciences, University of Alabama at Birmingham, Birmingham, USA; 5 Paraclinical Department, Faculty of Medicine, General Sir John Kotelawala Defense University, Ratmalana, Sri Lanka

**Keywords:** Peripheral arterial disease, Case control study, Risk factors, Sri Lanka

## Abstract

**Background:**

Peripheral artery disease (PAD) is an important global health problem and contributes to notable proportion of morbidity and mortality. This particular manifestation of systemic atherosclerosis is largely under diagnosed and undertreated. For sustainable preventive strategies in a country, it is mandatory to identify country-specific risk factors. We intended to assess the risk factors of PAD among adults aged 40–74 years.

**Methods:**

This case control study was conducted in 2012–2013 in Sri Lanka. Seventy-nine cases and 158 controls in the age group of 40–74 years were selected for the study in order to have case to control ratio 1:2. The criterion for selecting cases and control was based on Ankle brachial pressure index (ABPI). Cases were selected from those who had ABPI 0.85 or less (ABPI ≤0.85) in either lower limb. Controls were selected from those ABPI score between 1.18 and 1.28 in both lower limbs. Only newly identified individuals with PAD were selected as cases. Controls were selected from the same geographical location and within the 5 year age group as cases.

**Results:**

The history of diabetes mellitus more than 10 years (OR 5.8, 95% CI 2.2–14.2), history of dyslipidemia for more than 10 years (OR 4.9, 95% CI 2.1–16.2), history of hypertension for more than 10 years (OR 3.8, 95% CI 1.8–12.7) and smoking (OR 2.9, 95% CI 1.2–6.9), elevated HsCRP (OR 3.7, 95% CI 1.2–12.0) and hyperhomocysteinemia (OR 3.0, 95% CI 1.1–8.1) were revealed as country specific significant risk factor of PAD.

**Conclusions:**

Diabetes mellitus, hypertension, dyslipidemia, smoking as well as elevated homocysteine and HsCRP found as risk factors of PAD. Longer the duration or higher level exposure to these risk factors has increased the risk of PAD. These findings emphasis the need for routine screening of PAD among patients with the identified risk factors.

## Background

Peripheral artery disease (PAD) is an important global health problem and associated with considerably high morbidity and mortality [1]. It is a disease process resulting from obstruction of large peripheral arteries, exclusive of the coronary and intracranial cerebrovascular system, commonly due to atherosclerosis [2]. This chronic slowly progressive disease is usually characterized by occlusion of lower limb arteries ultimately causing acute or chronic limb ischemia. Although the association of PAD with higher risk of ischemic events has been identified, this particular manifestation of systemic atherosclerosis is largely under diagnosed and undertreated [2, 3]. The main systemic atherosclerotic vascular diseases, namely coronary artery disease (CAD), cerebrovascular disease (CVD) and PAD are leading causes of morbidity and mortality and all these diseases share the common pathophysiological process of atherothrombosis [4].

Advanced age, family history, smoking, diabetes mellitus, hypertension and dyslipidemia are commonly identified traditional cardiovascular risk factors of PAD [5–7]. A number of “nontraditional” risk factors for PAD have also been recognized including race and ethnicity, elevated inflammatory markers such as C-reactive protein, fibrinogen, leukocytes and interleukin-6, genetics, hypercoagulable states of altered blood levels of D-dimer, homocysteine, lipoprotein, and an abnormal waist-to-hip ratio [8]. The risk-factor identification is important because PAD is associated with reduction in functional capacity and quality of life as well as increased cardiovascular morbidity and mortality from myocardial infarction and CVA [9]. It is also associated with personal, social, and economic burden [3]. The risk factor modification plays an important role in managing patients with PAD in primary care setting and prevention of its complications [10]. Early diagnosis of PAD is essential to improve quality of life, to prevent further functional impairment, and to reduce mortality and morbidity from CAD and CVD. For sustainable preventive strategies in a country, it is mandatory to identify the prevalence of the disease and identifying country-specific modifiable risk factors. A recent study found the age and sex adjusted prevalence of PAD in Sri Lanka to be as 3.6% [11]. There are no studies on risk factors of PAD in Sri Lanka. This study was intended to identify the country specific risk factors of PAD which will help to address preventive measures of PAD in Sri Lanka.

## Methods

### Study population

This case control study was conducted in parallel to a cross sectional prevalence survey in the Gampaha district in Sri Lanka in 2013. Gampaha is the second most populous district in Sri Lanka, and has a population of 2 million. Cross sectional survey was conducted using a multistage probability proportionate to size sampling technique to recruit 2912 adults aged 40–74 years from 104 clusters. Cluster size was 28 with equal number of males and females. Detailed methodology of this cross sectional survey has been described in previously published paper [11]. Identification of cases and controls was based on ankle brachial pressure index (ABPI). The measurement of ABPI was performed according to the procedure described in American College of Cardiology and American Heart Association guidelines for the management of patients with peripheral arterial disease [6].Assessment of ABPI was done using “Summit Vista ABI L 450’’ arterial doppler instrument. The ABPI was calculated up to 2 decimal places for each lower limb as the ratio of the highest systolic blood pressures at the ankle and the highest of the left and right brachial systolic pressures. The cutoff level of ABPI to select cases and controls was based on a study validating ABPI measurements for Sri Lankan population [12]. To ensure 100% specificity and no false positives among cases, only newly identified individuals who had ABPI value 0.85 or less in either lower limb were selected as cases. There by, all those who were identified as cases were incident cases. Controls were identified from those who had ABPI within the range of 1.18–1.28 in both lower limbs. Those who had undergone any type of surgery or procedure for PAD previously were excluded from the study.

Sample size was determined based on the formula for case control study [13]. Odds ratios and rate of exposure among controls were obtained from both local studies and studies done in other countries [6, 14–16]. A confidence interval of 95% and a power of 80% were selected. Largest sample size was selected after applying reported odds ratios as the relative frequency of the exposure, assuring adequate precision and power [17]. To accommodate for low number of cases, case to control ratio was decided as 1:2, without compromising the power of the study [17, 18]. Thus, 79 cases and 158 controls were recruited for the study. Controls were selected from the same geographical location and within the 5 year age group as cases. It is well known that PAD is mainly an age related disease [19, 20]. Matching was done only for age variable. Thus, it allows all other selected variables to be assessed as potential risk factors of PAD.

### Data collection

Both cases and controls were subjected to an interviewer administered questionnaire (IAQ). IAQ inquired on socio demographic factors such as age, religion, ethnicity, level of education and family income. Presence of selected non-communicable disease conditions such as diabetes mellitus, hypertension, dyslipidemia, coronary artery disease, cerebrovascular disease and PAD was obtained with duration of illness. All these information were self-reported and verified by available clinical records or medicines. In addition, information on family history of selected non-communicable diseases; diabetes mellitus, hypertension, dyslipidemia, coronary artery disease, cerebrovascular disease and PAD and information on smoking, usage of alcohol, were also inquired. Smoking status was categorized as ever smokers, current smokers, and former smokers according to the classification of the Centers for Disease Control and Prevention in the United States [18]. Lifetime exposure to smoking was assessed by pack-year smoking values. Alcohol intake was categorized as abstainers, less frequent users and frequent users according to the definition of the National Institute on Alcohol Abuse and Alcoholism in United States [21]. In all participants generalized obesity was assessed using body mass index (BMI) and abdominal obesity was assessed using waist circumference (WC) and waist hip ratio (WHR). Body weight was measured in kilogram to the nearest 0.1 kg using a “Seca 876” electronic digital standing-on weighing scale. The standing body height measured in centimeter to the nearest 0.5 cm using a “Seca 213” stadiometer. The waist circumference and hip circumference measured in centimeters to the nearest 0.2 cm using a “Seca 201” ergonomic circumference measuring tape. Measurement of height, weight, waist circumference (WC) and hip circumference (HC) were carried out according to the guidelines given by anthropometry procedures manual, Centres for Disease Control in United States [22]. The cut off values for generalized obesity and abdominal obesity was defined based on the guidelines given by the International Obesity Task Force-WHO for Asians [23], and report of WHO expert consultation on waist circumference and waist-hip ratio respectively [24].

Fasting plasma glucose, lipid profile, high sensitivity C reactive protein (HsCRP) and serum homocysteine was measured using standardized fully automated techniques in an internationally accredited laboratory in a private hospital, Sri Lanka. The laboratory quality control measures included routine external and internal quality control procedures at regular intervals. The standard reagents were used for biochemical analysis as per the manufacturer recommendations. Reagents packs used in the study were supplied by Siemens healthcare Diagnostics Inc, Newark, USA and Abbott Laboratories, Germany which were pre-validated and approved for respective analyzers.

Cutoff values for DM and dyslipidemia was based on guidelines given by World Health Organization Department of Non-communicable Disease Surveillance and Adult Treatment Panel III (ATP III) National cholesterol education programme [25]. The serum HsCRP level more than 3 mg/L was considered as elevated HsCRP and the serum homocysteine level more than 15 mol/L was considered as elevated homocysteine or hyperhomocysteinemia [26, 27].

The Ethics Review Committee of the Faculty of Medicine, University of Colombo granted the approval for the study. Informed written consent was obtained from all patients prior to participation.

### Statistical analysis

Analysis was done using Statistical Package for Social Science (SPSS). Bivariate analysis for the PAD and non-PAD by selected characteristics of respondents was done and differences between groups were analyzed with Pearson’s Chi square tests. Unconditional Multivariate logistic regression (MLR) analysis was performed to identify the independent risk of each variable with PAD, adjusted by confounders. The model produced logistic regression co-efficient (β co-efficient), which estimated the adjusted OR for each of the independent variable in the MLR. The dependent variable used in the MLR analysis was the presence (cases) or absence (controls) of PAD. The independent variables used in the MLR analysis were based on the variables that showed a statistical significant association with PAD at a significance level of 0.05 in the bivariate analysis (unadjusted by confounding variables). Out of these variables, some variables were excluded from the MLR analysis if they had a limited number of subjects in one cell or if they showed a high inter-correlation with other variables. The independent factors included in the model were selected socio-demographic measures, presence of long term medical conditions such as DM, hypertension, dyslipidemia, smoking and alcohol usage, BMI, WC, WHR, presence of hyperhomocysteinemia and high level of HsCRP. Statistical significance was set at p < 0.05. MLR analysis was performed for identified dependent and independent variables by carrying out backward stepwise binominal LR method. The Probability for this method was fixed for entry at 0.05 and removal at 0.1 significance level. The model that showed the best goodness-of-fit in the MLR analysis was selected as the best model. Goodness of fit of the MLR was assessed by the overall percentage of the predictions that were correctly classified by observed outcomes. Goodness of fit of the MLR model was assessed using Omnibus tests, Hosmer and Lemeshow tests and Cox and Snell R square (R^2^) and Negelkerke R^2^ tests. The Omnibus tests of model coefficients were used to test how well the model performs or the capability of all predictors in the model jointly to predict the dependent variable. A well-fitting model is significant at the level of 0.05 or below, indicating that the final model fits the data adequately. Hosmer and Lemeshow test was also used as a goodness of fit test and good fit is significant at the level of 0.05 or more. Cox and Snell R^2^ and Negelkerke R^2^ values were also used to provide an indication of the amount of variation in the dependent variable explained by the model. The variables retained in the MLR model were considered as independent risk factors adjusted for the confounders for prediction of development of PAD.

## Results

All the selected cases (79) and controls (158) participated in the study making the response rate 100%. The mean age of cases was 64.3 years (SD 7.6) and median age was 65 years (IQR 60–70 years). The mean and median ages of controls were 64.3 years (SD 7.7) and 66 years (IQR 60–71 years) respectively.

Comparison of socio-demographic characteristics among cases and controls is shown in Table 1. Compared to controls a significantly higher proportion of cases had a history of diabetes mellitus (71.6%), hypertension (78.4%) and dyslipidemia (73.9%). When considering the duration of the disease, a significantly higher proportion of cases found to have history of 5 years or more for diabetes mellitus, hypertension, or dyslipidemia than controls (p < 0.01) (Table 2). Current smokers among cases (20.2%) were significantly higher than controls (8.2%) (p < 0.01). Further, the proportion of those exposed to 10 or more pack years among cases (31.6%) was also significantly higher than the proportion of controls (6.9%, p < 0.01) (Table 3).

**Table 1:** Bivariate analysis of demographic and socioeconomic characteristics

Demographic characteristics	Cases (n = 79)		Controls (n = 158)		p value
	No	%	No.	%	
*Age group (years)*					*
45	05	6.3	10	6.3	
50	07	8.9	14	8.9	
55	07	8.9	14	8.9	
60	16	20.3	32	20.2	
65	17	21.5	34	21.5	
70–74	27	34.2	54	34.2	
*Sex*					0.195
Male	39	49.4	92	58.2	
Female	40	50.6	66	41.8	
*Sector of residence*					0.401
Rural	56	70.9	120	75.9	
Urban	23	29.1	38	24.1	
*Ethnicity*					0.802
Sinhala	78	98.7	157	99.4	
Tamil	01	1.3	01	0.6	
*Level of education*					0.225
Up to Grade 10	37	46.8	61	38.6	
GCE O/L completed and above	42	53.2	97	61.4	
*Monthly household income Rs*					0.263
≥30,000	29	36.7	70	44.3	
<30,000	50	63.3	88	55.7	

* Not analyzed as age was matched for 5 year age groups

**Table 2:** Bivariate analysis of history of selected diseases

Disease	Cases (n = 79)		Controls (n = 158)		p value
	No	%	No	%	
*Diabetes mellitus by history*					
No history	22	27.9	109	69.0	<0.0001
<5 years	01	1.3	07	04.4	
5–10 years	11	13.9	10	6.3	
≥10 years	45	56.9	32	20.3	
*Hypertension by history*					
No history	18	22.8	86	54.4	<0.0001
≤5 years	04	5.1	27	17.1	
5–10 years	12	15.2	12	7.6	
≥10 years	45	56.9	33	20.9	
*Dyslipidemia by history*					
No history	22	27.9	95	60.2	<0.0001
<5 years	04	5.0	22	13.9	
5–10 years	20	25.3	17	10.7	
≥10 years	33	41.8	24	15.2	
*Presence of family history*					
Diabetes mellitus Yes	29	36.7	46	29.1	0.235
Hypertension Yes	25	31.6	49	31.0	0.921
Dyslipidemia Yes	20	25.3	22	13.9	0.030
CAD Yes	11	13.9	12	7.5	0.120
CVA Yes	16	20.3	18	11.4	0.066
PAD Yes	01	1.3	03	1.9	0.858

*CAD* coronary artery disease; *CVA* cerebrovascular disease; *PAD* peripheral arterial disease

**Table 3:** Bivariate analysis of smoking and alcohol usage

Smoking status	Cases (n = 79)		Controls (n = 158)		p value
	No	%	No	%	
Never smoker	49	62.0	121	76.6	0.018
Current smoker	16	20.2	13	8.2	
Former smoker	14	17.7	24	15.2	
*Type of smoking*					
Never smoker	49	62.0	121	76.6	0.080
Cigarette only	17	21.5	24	15.2	
Cigarette and Beedi	07	8.8	09	5.7	
Beedi only	6	7.6	04	2.5	
*Pack year smoking*					
<5	50	1.3	16	10.1	0.080
5	04	5.0	10	6.3	
≥10	25	31.6	11	6.9	
*Alcohol usage*					
Abstainers	42	53.2	97	61.4	0.071
Less frequent users	12	15.2	21	13.3	
Frequent users	25	31.6	40	25.3	

Anthropometric measurements were not found significantly different between cases and controls (Table 4). Two serum biomarkers were assessed to determine the risk of PAD. Based on distribution of serum values of HsCRP and homocysteine in controls, both cases and controls were categorized into quartiles. Homocysteine and HsCRP levels among PAD cases was significantly higher than controls (p < 0.001) and elevated blood levels of HsCRP (>3 mg/dl) and homocysteine (>15 mg/dl) than controls (p < 0.05) (Table 5).

**Table 4:** Bivariate analysis of anthropometric characteristics

	Cases (n = 79)		Controls (n = 158)		p value
	No	%	No	%	
*Generalized obesity*					
BMI<18.5 kg/m^2^	17	21.5	16	10.1	0.067
BMI 18.5–24.9 kg/m2	25	31.6	52	32.9	
BMI ≥25 kg/m2	37	46.8	90	57.0	
*High Waist circumference*					0.782
Yes	37	46.8	77	48.7	
No	42	53.2	81	51.3	
*High Waist hip ratio*					0.331
Yes	49	62.1	108	68.3	
No	30	37.9	50	31.6

**Table 5:** Bivariate analysis of high sensitivity C reactive protein and homocysteine

Biochemical parameter	Cases(n = 79)		Controls(n = 158)		p value
	No	%	No	%	
*High sensitivity CRP (HsCRP)*					
1st quartile	10	12.7	39	24.7	<0.001
2nd quartile	11	13.9	40	25.3	
3rd quartile	12	15.2	40	25.3	
4th quartile	46	58.2	39	24.7	
*Homocysteine*					
1st quartile	05	6.3	39	24.7	<0.001
2nd quartile	17	21.5	40	25.3	
3rd quartile	21	26.6	40	25.3	
4th quartile	36	45.6	39	24.7	
*Elevated Hs CRP (*>*3* *mg/dl)*					<0.001
No	32	40.5	118	74.7	
Yes	47	59.5	40	25.3	
*Hyperhomocysteinemia (*>*15* *mg/dl)*					<0.001
No	22	27.8	80	50.6	
Yes	57	72.2	78	49.4	

The statistical significance of the overall model as well as the significance of individual steps of the model was assessed by Omnibus test. In Omnibus tests of model coefficients, the Chi square value was 159.3. This value was highly significant (p < 0.001). Hosmer and Lemeshow test also supported the model, where Chi square value was 8.9 with 8 degrees of freedom (p = 0.35). Since poor fit is indicated by significance value <0.05, significance value 0.35 in this test support for the model. In the final model Cox and Snell R^2^ was 0.680 and the Nagelkerke’s R^2^ was 0.780. This means that the final model explains 68.0–78.0% of the variance in PAD in the sample. In logistic regression analysis the history of diabetes mellitus more than 10 years (OR 5.8, 95% CI 2.2–14.2), history of dyslipidemia for more than 10 years (OR 4.9, 95% CI 2.1–16.2), history hypertension for more than 10 years (OR 3.8, 95% CI 1.8–12.7) and ever smoking (OR 2.9, 95% CI 1.2–6.9) were found as risk factors. In addition, elevated HsCRP (OR 3.7, 95% CI 1.2–12.0) and hyperhomocysteinemia (OR 3.0, 95% CI 1.1–8.1) were also revealed as significant risk factor of PAD after controlling for other confounding factors (Table 6).

**Table 6:** Parameter estimates and their significance—multivariate logistic regression analysis

Variable	Category	OR	95% CI for OR	
			Lower	Upper
Diabetes mellitus by history	No	1.0		
	5–9 years	1.5	0.2	9.0
	≥10 years	5.8	2.2	14.2
Dyslipidemia by history	No	*1.0*		
	5–9 years	2.6	0.7	8.8
	≥10 years	4.9	2.1	16.2
Hypertension by history	No	*1.0*		
	5–9 years	2.6	0.7	8.8
	≥10 years	3.8	1.8	12.7
Smoking status	Never smoker	*1.0*		
	Ever smoker	2.9	1.2	6.9
ElevatedHsCRP	No (≤3 mg/l)	*1.0*		
	Yes (>3 mg/ml)	3.7	1.2	12.0
Hyperhomocysteinemia	No (≤15 mg/ml)	*1.0*		
	Yes (>15 mg/ml)	3.0	1.1	8.1

## Discussion

This is the first case control study on PAD in Sri Lanka. This study was able to identify country specific risk factors of PAD which will help to address preventive measures of PAD in Sri Lanka. Use of validated ABPI cut off values in selecting cases and controls minimized any misclassification in our study. Selecting non diseased individuals as cases as well as selecting disease individuals as control leads to under estimation of the risk [28]. The cutoff levels of ABPI for selection of cases and controls were based on the finding of a validation study of ABPI [12]. All the cases of our study were incident cases detected during the community based prevalence survey [11]. As latent period of PAD is long, modification of risk factors following diagnosis becomes a major drawback if prevalent cases were selected for a case control study. Hence, we were able to minimize differential recall of possible exposure to risk factors between cases and controls in our study by only enrolling incident cases.

The final MLR model was able to predict 68–78% of the variance of PAD, indicating that over two-thirds of the variables determining PAD have been identified in our study. Thus, findings of this study are useful for healthcare providers to take preventive action to reduce the burden of disease in the country by early identification of risk factors. In the multivariate analysis, presence of history of diabetes mellitus, hypertension or dyslipidemia for 10 or more years, smoking, elevated CRP (>3 mg/dl), hyperhomocysteinemia (>15 mg/dl) were found to be independent risk factors for PAD among Sri Lankan adults. There are number of supportive research findings similar to our study. American Diabetes Association reported diabetes mellitus as the strongest risk factor of PAD [29]. National Health and Nutrition Examination Survey found Odds ratio (OR) of 2.08 for presence of DM (95% CI 1.01–4.28) [30]. In the San Diego population study the OR of diabetes mellitus for PAD was reported as 6.9 (p < 0.001) [19]. Dyslipidemia also has been identified as a significant risk factor for PAD in many studies. Ridker et al. reported dyslipidemia as an independent risk factor (RR 3.0, 95% CI 1.5–6.1) [31]. National Health and Nutrition Examination Survey also found the OR for dyslipidemia as high as 1.7 (95% CI 1.01–2.74) [30]. A cross sectional study conducted in Finland by Korhonen et al.(OR 2.3, 95% CI 1.56–6.58), Rotterdam follow up study (OR 1.32, 95% CI 1.07–1.64) and San Diego Population Study (RR 1.85, p = 0.01) have reported hypertension as a significant risk factor of PAD [19, 32, 33].

Most of the previous studies have assessed the risk for PAD only in relation to the presence or absence of diabetes mellitus, dyslipidemia and hypertension and not related to the duration of the illness. Although the exact time of onset of these conditions is difficult to estimate, at least the known duration of disease after detection provides important information about development of PAD. Therefore, we assessed the risk of PAD according to the history of duration of diabetes mellitus, dyslipidemia and hypertension. Having diabetes mellitus, dyslipidemia or hypertension for 10 years or more duration was found as significant risk factors of PAD. Korhonen et al. [32], reported that newly diagnosed pre-diabetes or diabetes per se is not associated with PAD, whereas long-lasting diabetes mellitus remains a well-established risk factor. In a prospective cohort study in USA, Joosten et al. [34] highlight that incidence of PAD significantly increases with the duration of diabetes mellitus, dyslipidemia and hypertension.

We found that ever smoking as significant risk factor for PAD in the MLR analysis. Many studies have identified smoking as a potent risk factor of PAD with consistent dose response relationship. A study carried out in Beijing, found both current smoking and former smoking (OR 1.5, 95% CI 1.1–2.1) as a significant risk factor of PAD [35]. The San Diego Population Study has identified pack year smoking more than 20 (OR 1.6, p < 0.01) and Framingham offspring study-pack year smoking of 10 years (OR 1.3, CI 1.2–1.4) as significant risk factors of PAD [19, 36]. A systematic review by Willigendael et al. found that the prevalence of PAD had increased by 2–3-fold among current smokers compare to nonsmokers and a clear dose–response relationship [37]. In our study pack year smoking was not found as a significant risk factor in the final MLR model. No significant association was found between use of alcohol and PAD in our study. Similar results have been reported in previous studies as well [33–36]. However, Strong heart study found that current alcohol consumption is negatively associated with PAD (OR 0.26, p < 0.03) [38]. However, it was based on a minority population in USA. Therefore results of Fabsitz et al. study cannot be considered conclusive.

We assessed the association between blood levels of HsCRP and homocysteine with PAD. In the present study we found elevated HsCRP (>3 mg/L) and Hyperhomocysteinemia (>15 mg/dl) to as significant risk factors of PAD. Many previous case control and follow up studies [31, 39, 40], have identified higher level of HsCRP as a significant risk factor of PAD. Garofolo et al. in a cross sectional study in Brazil [41], found that the risk of homocysteinemia for PAD was significant (OR 1.5, 95% CI 1.02–2.25). Aronow and Ahnalso reported homocysteine as a significant risk of for PAD (OR 1.12, p < 0.001) [42]. Systematic reviews carried out by Boushey et al., [43] and Khandanpour et al., [44] also found homocysteine as a significant risk factor of PAD (OR 4.3, 95% CI 1.7–6.9) supporting our results.

Literature shows inconsistent results for sex as a risk factor of PAD. Bennett et al. reported that males are at significant higher risk for PAD among migrant South Asians in UK (OR 2.81, 95% CI 1.31–6.0). In the same study they found sex was not a significant risk factor among black ethnicity the (OR 0.65, 95% CI 0.21–2.01) [45]. In contrary, Sigvant et al. found a higher prevalence of PAD among females [46]. Similar to our findings, many studies has reported non significant association between sex and PAD [19, 30]. Similarly, way inconsistent results have been reported by many studies for obesity as a risk factor of PAD. In our study BMI, HR and WHR were not found as significant risk factors of PAD. The Beijing study, reported that risk for PAD was 1.05 (95% CI 1.02–1.08) for each 1 kg/m^2^- increment in BMI [35]. Lu et al. has found that WHR increases the risk of PAD in men (OR 4.68, 95% CI 2.1–10.3) and higher WC increases the risk of PAD in women (OR 2.94, 95% CI 1.01–8.8) [47]. Some studies have reported BMI as a protective factor for PAD [19, 48]. However, many others reported the association between obesity and PAD was not significant [30, 33, 36, 49–53]. It should also be noted that findings in studies conducted in different populations, particularly in minority groups or in diverse settings has shown slight differences. Thus, association of obesity and sex with PAD was inconclusive.

## Conclusion

These results demonstrate diabetes mellitus hypertension or dyslipidemia as well as smoking as country specific risk of PAD. In addition, there is a high risk of PAD among persons with high level of homocystein and HsCRP. Longer the duration or higher level exposure to these risk factors has increased the risk of PAD. These findings emphasis the need of routine screening of PAD among individuals with the identified risk factors.

## Abbreviations

ABPI: ankle brachial pressure index
BMI: body mass index
CAD: coronary artery disease
CVD: cerebrovascular disease
PAD: peripheral arterial disease
HsCRP: high sensitivity C reactive protein
IAQ: interviewer administered questionnaire
WC: waist circumference
WHR: waist hip ratio
